# Thirty-Fifth Annual Meeting of the Mississippi Valley Association of Dental Surgeons

**Published:** 1879-06

**Authors:** Edward G. Betty


### Proceedings of Societies.

### THIRTY-FIFTH ANNUAL MEETING OF THE MISSISSIPPI VALLEY ASSOCIATION OF DENTAL SURGEONS.

This association convened on Tuesday, March 5th, 1879, at ten o'clock a. m., in the Dental College Hall, President F. A. Hunter presiding. After prayer by Dr. Leslie, W. H. Sillito was elected secretary pro tem., the recording secretary being absent. The minutes of the last meeting were read and with correction, approved. Reports from committees being called for, the executive committee submitted the following programme for discussion:

1. Chemical processes that occur in the mouth.
2. Effects of systemic disease upon the dental organism.
3. The influences upon second dentition of the derangement of the first.
4. The new dental departure.
5. Dental education and professional culture.
6. Novel dental operations.
7. Modus operandi of topical remedies in inflammatory conditions of the dental organism.
8. Morbid conditions of the pulp, and how diagnosed.

On motion the report as embodied in the programme for discussion, was accepted by the association. The treasurer, Dr. Cameron, read his report, which, upon acceptance, was handed to Drs. N. S. Hoff and W. H. Sillito, the appointed auditors.

On motion of Dr. J. Taft the morning sessions were fixed at from nine to twelve o'clock, and the afternoon from half-past one to half-past five o'clock. Dr. Berry made his annual motion prohibiting the smoking of tobacco in the building during meeting hours; carried. On motion of Dr. J. Taft the final adjournment was fixed at twelve o'clock m., on Fric day, unless otherwise ordered. The committee on membership reported Dr. C. H. Rosenthal, of Cincinnati, Ohio, as a candidate for admission, who, upon motion, was elected by the secretary's ballot. Dr. D. C. Whaley, of Pomeroy, O., having been a member some ten years ago, was reinstated upon payment of two dollars.

The first subject on the programme was now taken up, Dr. J. Taft leading the van, making a comprehensive introduction of the topic. Dr. Cassidy followed, giving many of the chemical re-actions which take place. Drs. H. A. Smith, Morgan, Leslie and Harriott all participated in the discussion. Adjourned till half past one o'clock.

FIRST DAY—AFTERNOON SESSION.

President Hunter called the association to order at two o'clock. Minutes of morning session were read. On motion of Dr. J. Taft, each member was allowed fifteen minutes for his first speech and five minutes for each subsequent one. The first subject being finished, the second in order was started by Dr. J. Taft, followed successively by Drs. Rawls, Meredith, Smith, Osmond, Jas. Taylor, Morgan and Templeton.

Miss Dr. Howells, a practicing physician of Cincinnati, was on motion, accorded the privileges of the floor, the same were also extended to all visitors present not members of the association.

The third subject for consideration was then taken up, Dr. J. Taft again coming to the front and opened the way for Drs. Morgan, G. W. Keely, Smith, Berry, Rawls, Osmond and Clayton, by whom the discussion was continued till adjournment.

SECOND DAY—MORNING SESSION.

Meeting came to order at ten o'clock. President Hunter presiding. The minutes of the previous day's afternoon session were read and approved. The auditing committee reported that the treasurer's report was correct, which upon motion was accepted, and the committee discharged.

The discussion of "The Influences upon Second Dentition of the Derangement of the First," was resumed by Drs.

Molyneaux, Dennis, G. W. Keely and J. Taft, making the concluding remarks upon the subject.

The fourth topic in order being announced, vice-president J. W. Jay assumed the duties of the chair, and Dr. J. Taft started the talk. Dr. H. A. Smith read the completed portion of an unfinished paper upon the subject; Drs. Leslie, Smith, Craven, Berry, Carter, Sage and Osmond, carrying on the discussion till adjournment. The afternoon of this day was devoted to the exercises of the Ohio Dental College Alumni Association.

### THIRD DAY—MORNING SESSION.

Meeting called to order at ten o'clock, by President Hunter. Minutes of previous session read and approved. Miscellaneous business being called for, Dr. Cassidy moved that the secretary be instructed to cast affirmative ballot for the admission of the following gentlemen to membership, viz.:—Drs. J. H. Prewitt, Ky.; A. G. Rose, O.; Hart Cameron, O.; and O. N. Heise, O.; so ordered.

The discussion of the fourth topic was resumed, Drs. How, Smith, Leslie and J. Taft completing it. Dr. Jay moved that the fourth subject be passed, as also the fifth and sixth, in order that the seventh might be reached. Carried.

On motion of Dr. J. Taft this action was reconsidered and thirty minutes devoted to discussing the sixth subject. Dr. Osmond made the preliminary remarks, Drs, French, How, Hunter, Berry, Templeton, Welch, Wm. Taft and Jas. Taylor, taking part till the matter was closed by limitation.

Upon motion Drs. Morgan, Cassidy, J. Taft and Smith, were appointed as committee to draft resolutions upon the death of Prof. J. H. McQuillan.

The seventh subject on the programme was taken up for thirty minutes' discussion, Drs. Cravens, Jay, Rawls, Jas. Taylor, J. Taft and Morgan participating.

The following resolutions were then reported and read:

Whereas, the members of the Mississippi Valley Dental Association, have heard with profound sorrow of the recent sudden death of Prof. John Hugh McQuillan, of Philadelphia.

"Therefore, *Resolved*, That by this visitation of Divine Providence, in the death of Prof. McQuillan, our profession, and indeed, the entire world of science, have lost one of the very brightest minds vouchsafed to our generation. Cut off in the prime of life, in the midst of most energetic and successful efforts in the cause of the highest dental progress, his death will leave a void in the domain of education impossible to fill.

"*Resolved*, That the Mississippi Valley Dental Association tenders its sincere sympathy to the afflicted family and to the faculty of the Philadelphia Dental College, in *their* irreparable loss.

"*Resolved*, That a copy of these resolutions be sent to the family of the deceased, to the Faculty of the Philadelphia Dental College, and recorded in the minutes of this association."

W. H. Morgan,

J. S. Cassidy,

J. Taft,

H. A. Smith,

Committee.

These resolutions were most heartily and unanimously adopted by the association.

Drs. S. C. Carter and J. S. Hacker were elected to membership by the secretary's ballot.

The election of officers for the ensuing year being in order, the polling staff was elected to serve in their respective places:

President, J. S. Cassidy.
First Vice-President, J. W. Jay.
Second Vice-president, J. W. Sage.
Treasurer, J. G. Cameron.
Recording Secretary, E. G. Betty.
Corresponding Secretary, N. S. Hoff.

Upon resigning the chair, the President, Dr. F. A. Hunter, with appropriate remarks, presented the association with a rosewood gavel, the silver plate upon which bore this inscription: "Mississippi Valley Dental Association, organized 1844." Drs. Clayton and Jay conducted to the chair the president-elect, who immediately proceeded to business by appointing the standing committees as follows:

Executive.—J. Taft, A. O. Rawls, Jas. Taylor,

Membership.—J. E. Cravens, H. M. Reid. C. J. Keely.

Publication.—H. A. Smith, J. R. Clayton, Wm. Taft.

Ethics.—J. A. Watling, J. H. Prewitt, Wm. Van Antwerp.

Bills amounting to $31.75 were ordered paid. Drs. G. W. Keely and J. G. Cameron, were appointed to nominate delegates to the American Dental Association, all of whom were elected, viz:

S. J, Way, J. W. Jay, C. H, Rosenthal, E. G. Betty, N. S. Hoff, J. E. Cravens, J. F. Dennis, J. G. Cameron, J. S. Hacker, J. H. Prewitt, E. W. Anderson, C. I. Keely, Wm. Taft, W. S. How, E. C. Sloan, D. C. Whaley, J. R. Clayton, J. A. Stipp, A. Berry, R. G. Richter.

The association then adjourned to meet again at the same place on the first Tuesday in March, 1880, at ten o'clock, a. m.

EDWARD G. BETTY,
Recording Secretary.

